# Inhibition of succinate dehydrogenase activity impairs human T cell activation and function

**DOI:** 10.1038/s41598-020-80933-7

**Published:** 2021-01-14

**Authors:** Claudia Nastasi, Andreas Willerlev-Olsen, Kristoffer Dalhoff, Shayne L. Ford, Anne-Sofie Østergaard Gadsbøll, Terkild Brink Buus, Maria Gluud, Morten Danielsen, Thomas Litman, Charlotte Mennè Bonefeld, Carsten Geisler, Niels Ødum, Anders Woetmann

**Affiliations:** 1 LEO Foundation Skin Immunology Research Center & Department of Immunology and Microbiology, University of Copenhagen, Panum Institute, The Maersk tower, 07.12.76, Blegdamsvej 3C, 2200 Copenhagen, Denmark; 2 MS-Omics, Vedbæk, Denmark; 3 Department of Immunology and Microbiology, University of Copenhagen, Copenhagen, Denmark; 4 LEO Pharma A/S, Ballerup, Denmark

**Keywords:** Cell biology, Immunology

## Abstract

T cell activation is intimately linked to metabolism, as distinct metabolic requirements support the functional and phenotypical differences between quiescent and activated T cells. Metabolic transition from mitochondrial oxidative phosphorylation to aerobic glycolysis is crucial for a proper T cell activation. However, the role of tricarboxylic acid cycle (TCA), and in particular succinate dehydrogenase (SDH) in activated T cells needs further elucidation. Here we show that inhibition of SDH during activation of T cells results in strong impairment of proliferation, expression of activation markers, and production of key inflammatory cytokines, despite a concomitant increase in glycolytic metabolic activity. Similar effect of SDH inhibition were demonstrated in pre-activated T cell. Interestingly, itaconic acid, an endogenous SDH inhibitor released from activated macrophages and dendritic cells, had no immunomodulator effect. Taken together, our findings demonstrate that SDH enzyme fitness is critical for mounting and maintaining appropriate activation and function of human T cells.

## Introduction

Circulating naïve T cells are quiescent, but upon T cell receptor (TCR) stimulation, in combination with engagement of co-stimulatory molecules, they become activated. This, results in extensive proliferation, differentiation and acquisition of effector functions, such as the capacity to produce cytokines and other effector molecules^1,2^.

Functional and phenotypical differences between quiescent and activated T cells have distinct metabolic requirements^3–5^. Activated T cells undergo a metabolic transition from oxidative phosphorylation (OXPHOS) to glycolysis^6,7^. Signals from growth factors, like interlukin-2 (IL-2), and costimulatory molecules, like CD28, promote the switch to aerobic glycolysis, where glucose is fermented into lactate, independently of the mitochondrial tricarboxylic acid cycle (TCA), despite the presence of oxygen^8,9^: a process referred as the Warburg effect^7,10,11^. Importantly, it has been shown that T cell survival, proliferation and cytokine production are impaired in the absence of glucose, even with the presence of an alternative carbon source, such as glutamine^12,13^.

Most attention with regard to metabolic reprogramming in tumour cells and activated T cells has focused on the engagement and importance of aerobic glycolysis. However, recent publications have revealed a previously underrated relevance of mitochondrial-driven activities^14–17^, as was elegantly commented by DeBerardinis and Chandel^18^. In particular, succinate dehydrogenase (SDH) has attracted attention. SDH is part of the TCA, driving the enzymatic conversion of succinate into fumarate, and plays a major role in OXPHOS as electrons acceptor complex II. Interestingly, the enzymatic activity of SDH is required for optimal activation and terminal effector function of murine T helper 1 (Th1) cells, but in parallel suppresses Th1 proliferation and histone acetylation^15^. Moreover, inhibiting complex II or complex III resulted in reduced IFN-γ production^15^ and impaired T cell activation both in vitro and in vivo^17^. Likewise, more studies have upgraded TCA-derived metabolites from general intermediates of mitochondrial metabolism into the proper status of signalling molecules^19,20^. In particular, succinate seems to play multiple roles, thus to have hypertensive effect by modulating the renin-angiotensin system^21^, the ability to signal via GPR91 (SUCNR1) on dendritic-cells and increase the antigen specificity of activated T cells^22^, and the capacity to stabilize and activate HIF-1α in macrophages^23^. Lately, it has been demonstrated that the TCA-derived itaconic acid (ITA), which is produced via citrate accumulation in activated dendritic cells (DCs) and M1 macrophages^24,25^, has SDH inhibitory properties, that lead to the intracellular accumulation of succinate and modulation of cytokine secretion and oxygen consumption^26,27^. Additionally, it has been shown that activated macrophages secrete itaconic acid in the microenvironment, where it supresses bacterial growth, further supporting the role of ITA in innate immunity^28,29^. Nonetheless little is known regarding the effect of ITA on the adaptive compartment of the immune system.

Our findings highlight the vital importance of SDH as the key modulator in sustaining human CD4^+^ and CD8^+^ T cell activation, proliferation and functionality. In addition, we demonstrate that ITA has no effect on either endo-metabolome, gene expression profile or oxygen consumption in human T cells, while its derivatives DI and 4OI exhibit distinct capacities to modulate their activation and functionality.

## Results

### SDH inhibition impact proliferation, viability, and mitochondrial potential

**Figure 1:**
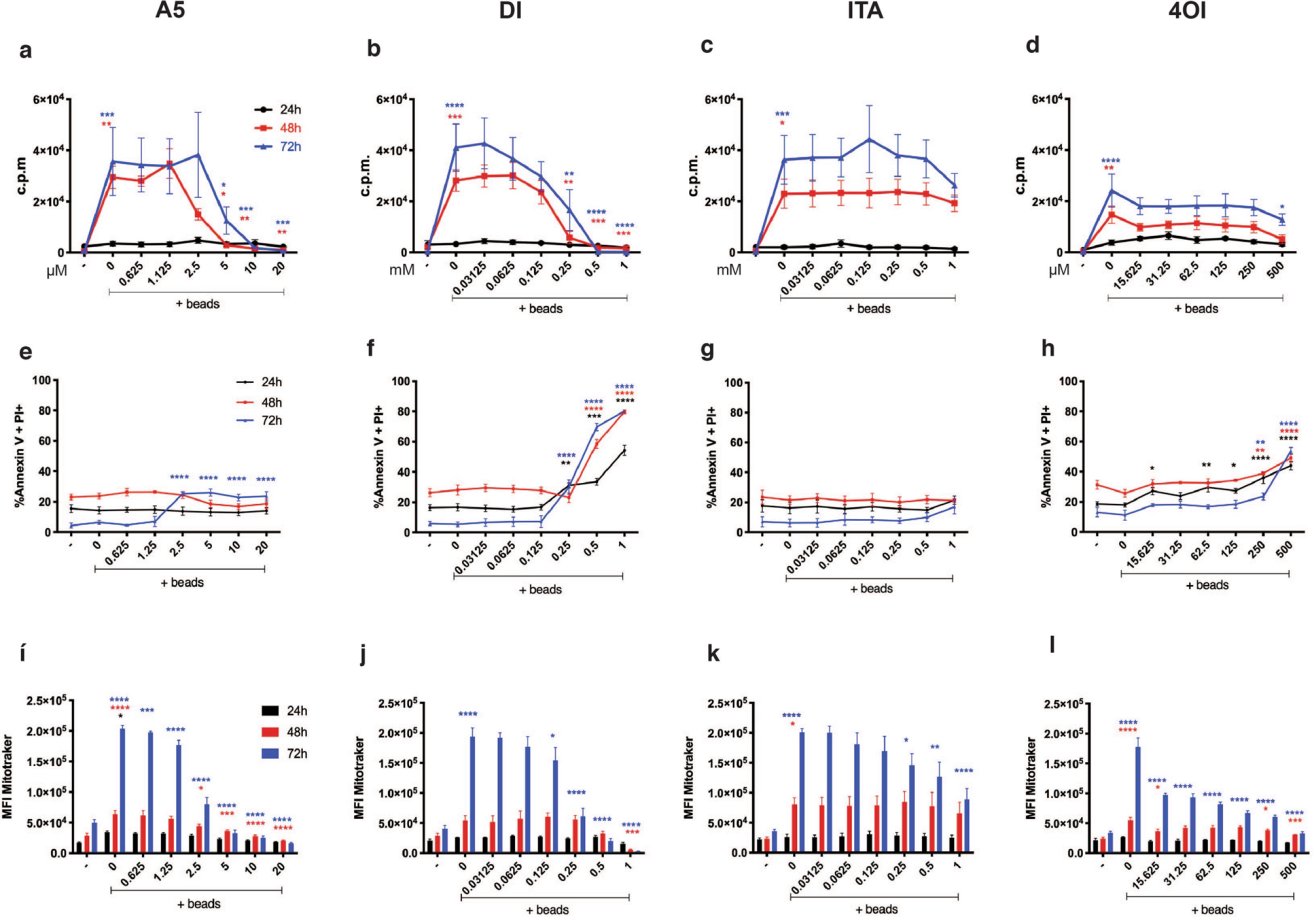
SDH inhibition impact proliferation, apoptosis, and mitochondrial potential. PBMCs were activated with anti CD3/CD28 beads and exposed to serially diluted atpenin A5 (A5), dimethyl-itaconate (DI), itaconic acid (ITA), or 4-octyl-itaconate (4OI). (**a**–**d**) Proliferation was evaluated via ^3^H-thymidine incorporation assays showed as c.p.m. (counts per minutes) (n = 3, with six technical replicates) (**e**–**h**) Percentages of dead cells are provided as double positive for annexin V and propidium iodide staining. (**i**–**l**) Mean Fluorescence Intensity (MFI) is shown after mitotracker staining gated out of live cells (n = 3 independent experiments). Continuous lines or bar charts were coloured referring to different time points: 24 h black, 48 h red, 72 h blue. All charts are representative of mean and ± s.e.m. P values are calculated using two-way ANOVA test followed by Dunnet’s test to the activated untreated control. *P < 0.05, **P < 0.01, ***P < 0.001, ****P < 0.0001.

To test whether the TCA cycle contributes to T cell fitness, human PBMCs were isolated and activated with anti-CD3/CD28 beads for 24 h, 48 h, and 72 h, alone or in presence of the SDH inhibitor atpenin A5 (A5), or putative inhibitors such as itaconic acid (ITA) and its analogues dimethyl-itaconate (DI), and 4-octyl-itaconate (4OI). T cell proliferation was evaluated using thymidine incorporation, and we found that the three highest concentrations of A5 impaired cells proliferation already after 48 h (Fig. 1a). Treatment with DI showed the same trend (Fig. 1b). After 48 h, 4OI reduced cell division significantly at the highest concentration, whereas lower concentrations homogenously weakened proliferation at 72 h (Fig. 1d). In contrast, ITA did not alter proliferation even after 72 h (Fig. 1c). In parallel, the cells were stained with annexin V and propidium iodide, and induction of cell death was analysed by flow cytometry. Results showed that A5 only marginally, and at very late time point, affected viability (Fig. 1e), while both DI and 4OI were effectively toxic at elevated concentrations already within 24 h (Fig. 1f,h, respectively). Again, ITA was confirmed to be ineffective, and triggered no consequences on viability (Fig. 1g).

Finally, we used mitotracker staining to check whether the mitochondrial potential of live cells (double negative for annexin V and PI) was influenced by each individual compound. Overall, we observed that activation of T cell induced a significant increase in mitochondrial membrane potential over time. Consistent with our previous data, we witnessed a reduced membrane potential in cells treated with the highest concentrations of A5, DI, or 4OI after 48 h (Fig. 1i,j,l). On the other hand, ITA did not perturb mitochondrial membrane potential except at the highest concentration, and only after 72 h (Fig. 1k).

### The role of TCA in sustaining T cell activation

**Figure 2:**
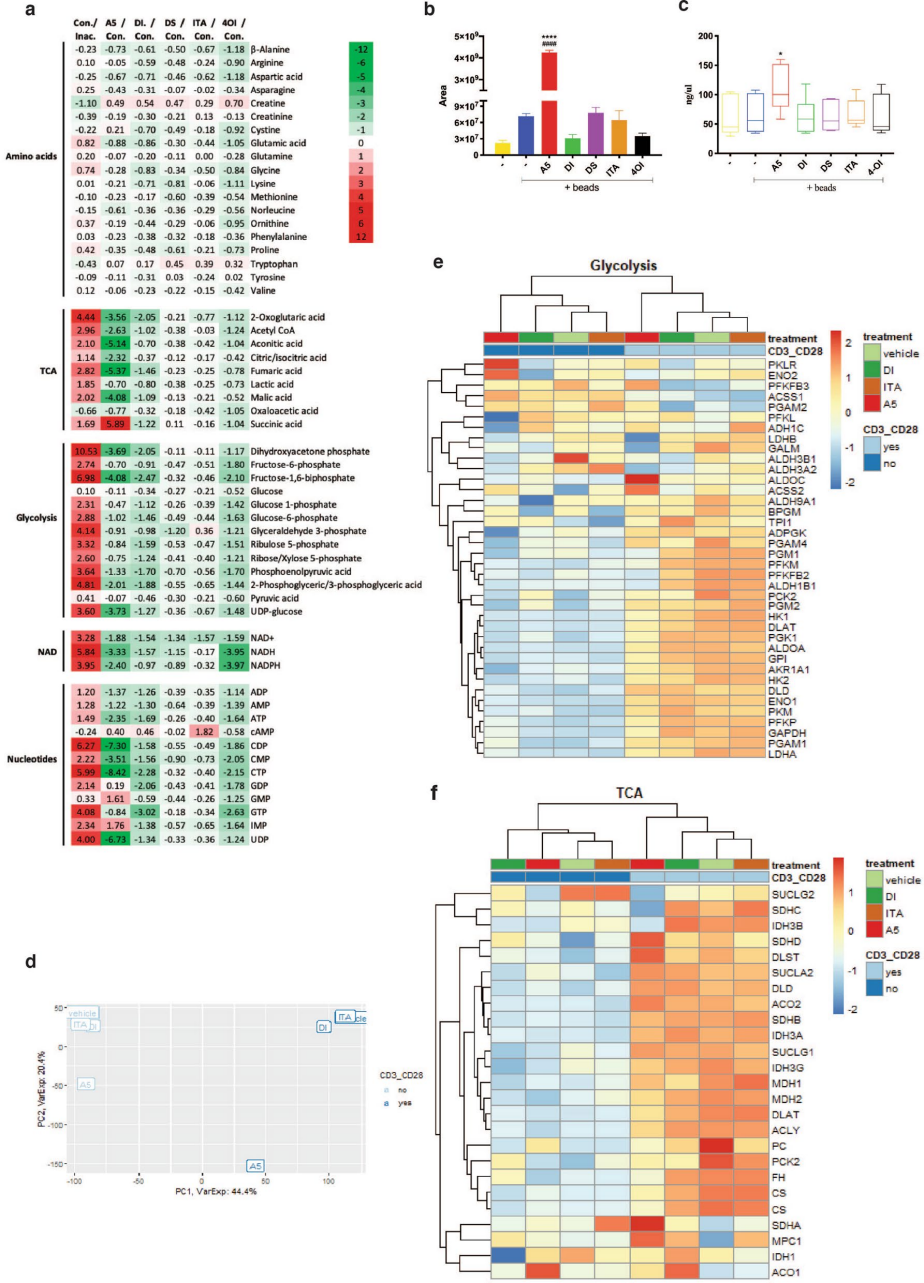
Role of TCA in human CD4^+^ T cells. Activated CD4^+^ T cells treated with A5 (20 μM), DI (250 μM), DS (1 mM), ITA (1 mM), or 4OI (125 μM). (**a**) Cellular metabolites measured by liquid-chromatography–mass spectrometry and shown as heat-map. Fold change values were obtained using the activated untreated cells as control (n = 3 independent experiments). (**b**) Succinic acid measured by LC–MS (n = 3 independent experiments) and (**c**) by Succinate Assay kit (Abcam) (n = 6, with n = 2 technical replicates each). (**d**) Visualization of the samples by principal component analysis plot (PCA) of resting and activated CD4^+^ T cells. (**e**, **f**) Heat maps with Z-score of TCA- and glycolysis-related genes from Affimetrix gene expression array performed on (pooled RNA from n = 3 experiments) resting and beads-activated CD4^+^ T cells exposed to A5, DI, or ITA. The bar blots are representative of mean and ± s.e.m (**b**) and boxes are representative of mean and 5–95 percentile. P values are calculated using one-way ANOVA test followed by Dunnet’s test to different control groups: (#) to the unstimulated and untreated control, and (*) to the activated untreated control. # or *P < 0.05, ## or **P < 0.01, ### or ***P < 0.001, #### or ****P < 0.0001.

Next, we aimed to investigate the efficacy of the selected putative inhibitors on the SDH enzymatic activity in human CD4^+^ T cell. We therefore performed endo-metabolome analysis by liquid chromatography–mass spectrometry (LC–MS) on human CD4^+^ T cells, activated for 24 h with anti-CD3/CD28 beads and treated with each individual compound at the time of the activation. In parallel, intracellular succinate concentration was quantified using a colorimetric assay method (Fig. 2a–c). For both assays we included samples treated with dimethyl-succinate (DS), trying to mimic the intracellular increase of succinate without depending on the enzymatic activity of SDH. To evaluate the results, we compared the values of the control (activated, untreated samples) to the inactive control (inactive, untreated samples), and then to each individual compound-treated sample (Fig. 2a–c). As expected, we found that the CD4^+^ T cell activation affected metabolism, resulting in a strong increase of glycolysis intermediates, underlining the primary role of glycolysis in supporting the functional switch^1,30^. Interestingly, also TCA intermediates were also highly upregulated, highlighting the supporting role of TCA in T cell activation (Fig. 2a–c, and Fig. S2a).

Among the tested compounds, only A5 showed true SDH inhibitory capacity resulting in increased levels of succinate (Fig. 2a–c, and Fig. S2b) [not mediated by the increase of intracellular itaconic acid (Fig. S2b)] and, as expected, reduced TCA metabolites downstream of SDH (Fig. 2a).

Intracellular detection of DI is not amenable to the electro-spray ionization LC–MS method used in these experiments; therefore, we cannot be sure that DI was internalized (Fig. S2c). In addition, DI did not increase itaconic acid levels or affect SDH activity (Fig. 2a–c and Fig. S2c). Furthermore, the addition of exogenous DS had no effect on intracellular succinate levels, but we observed an interesting increment of the mono-methylated form (methyl-succinic acid), indicating that DS is probably absorbed, modified by cellular esterases and partially enters the TCA (Fig. 2a–c, and Fig. S2d).

Surprisingly, we observed no changes in succinate levels in all the other samples, indicating that in CD4^+^ T cells ITA or its analogue 4OI does not inhibit SDH neither directly nor indirectly via increased concentration of intracellular itaconate (Fig. 2a–c and Fig. S2c–f). This is in contrast to previous observations made in macrophages, where ITA and analogues were shown to induce itaconate intracellularly and inhibit SDH^26,31,32^. Additionally, a recent publication has shown that only ITA and 4OI are capable of inducing intracellular itaconic acid accumulation, and exclusively ITA to be a SDH inhibitor^27^.

Furthermore, we investigated the effect of SDH inhibition on gene expression in resting and activated CD4^+^ T cells. The results supported the findings from the endo-metabolome assay, as visualised in principal component analysis plot (PCA) (Fig. 2d). Treatment with A5 affected gene expression in both resting and activated T cells, whereas ITA and DI had little, or no, effect when compared to vehicle control (Fig. 2d). Interestingly, A5 treatment induced the expression of SDH subunit genes (SDHA, SDHD, and SDHB) in activated T cells (Fig. 2f), likely as feedback response to compensate for the inhibition of the enzyme. In accordance, stimulation of T cells resulted in major changes in the gene expression profile including induction of both glycolysis- and TCA-related gene clusters (Fig. 2e,f), supporting a key role of TCA during T cell activation.

### SDH is pivotal for T cell activation

**Figure 3:**
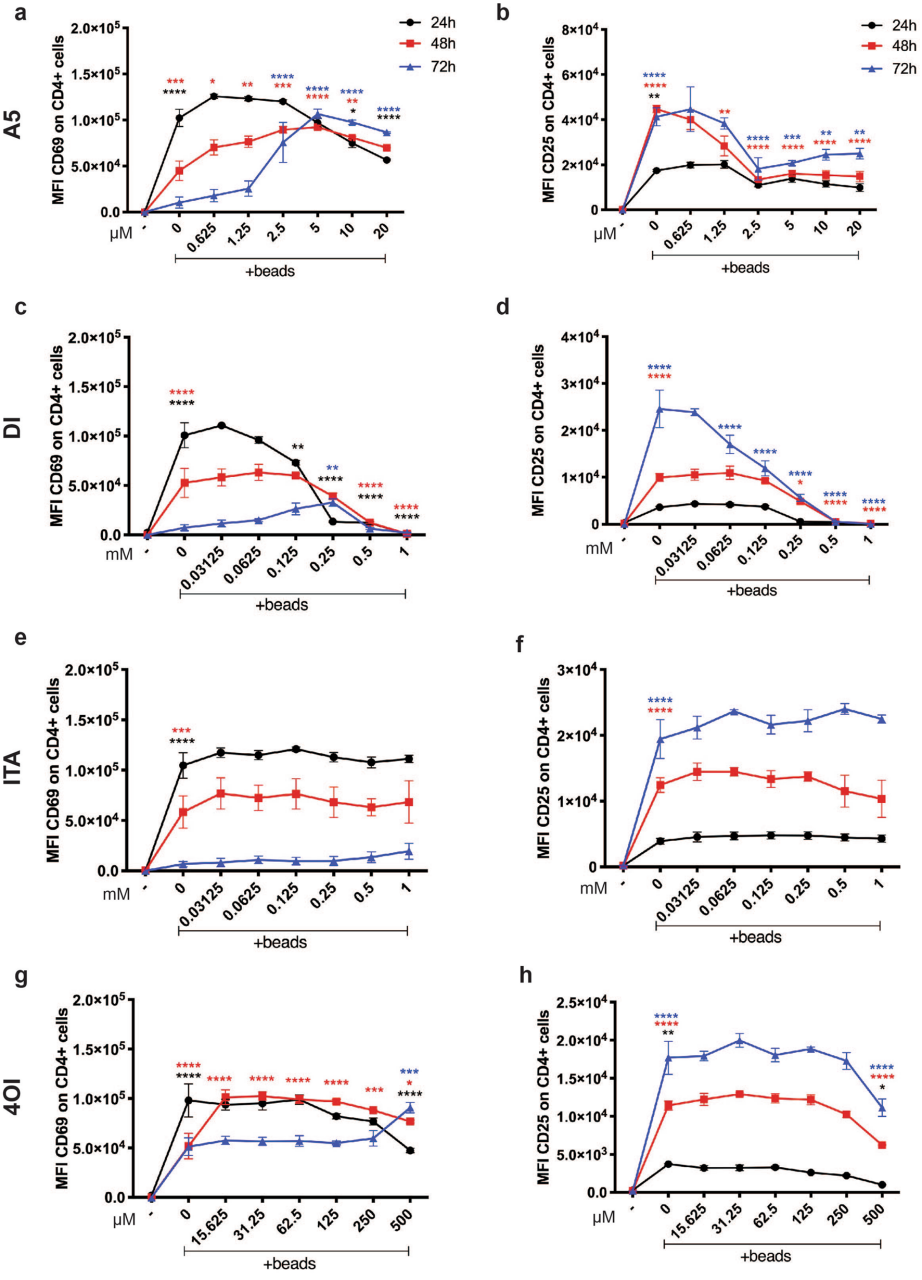
Flow cytometry analysis of CD4^+^ T cells surface activation marker CD25 and CD69. Isolated human CD4^+^ T cells were exposed to (**a**, **b**) A5, (**c**, **d**) DI (**e**, **f**), ITA, and (**g**, **h**), 4OI, in a concentration gradient and analysed after 24 h (black), 48 h (red), and 72 h (blue) by flow cytometry, gating on live cells. Mean Fluorescence Intensity (MFI) ± s.e.m. are shown for CD25 and CD69 expression levels (n = 3 independent experiments). P values are calculated using two-ay ANOVA. *P < 0.05, **P < 0.01, ***P < 0.001, ****P < 0.0001.

To test the contribution of SDH to T cell activation, we stimulated human PBMCs with anti-CD3/CD28 beads as before and treated them with increasing concentration of A5, DI, ITA or 4OI, and measure the surface expression of the classical activation markers CD69 and CD25 upon live CD4^+^ T cells, by flow cytometry (Fig. 3a–h and Fig. S1a–d). A5 strongly reduced surface expression of the early activation marker CD69 already within 24 h at the highest concentration, while the expression was slightly maintained after 48 and 72 h (Fig. 3a). CD25, which is usually expressed at later time points of activation, was strongly reduced by A5 within the 48 h, and 72 h (Fig. 3b). Accordingly, high concentrations of DI also reduced early CD69 expression from 48 up to 72 h (Fig. 3c) and had a profound inhibitory effect on CD25 expression already at 48 h, which was maintained up to 72 h (Fig. 3d). In contrast, ITA had no effect on the expression of either marker (Fig. 3e,f). 4OI marginally altered the expression of CD25 expression at the highest concentration (Fig. 3h), but significantly reduced early expression of CD69 at the highest concentration, while surprisingly seemed to induce expression (compared to control) after 48 h, and 72 h (Fig. 3g).

In parallel, we have also studied the CD8^+^ T cell compartment. We found that all compounds affected the expression of CD69 comparably to what was observed in CD4^+^ T cell (Fig. S1i–l). We expected, and observed, a slower induction of CD25 expression in CD8^+^ T cells (Ext. Data 1 m). ITA showed no pronounced effect on CD25 expression in CD8^+^ T cells (Fig. S1o), whereas 4OI only reduced CD25 expression at the highest concentration and after 72 h. Similarly to the CD4^+^ T cell data, A5 and DI strongly reduced CD25 expression in CD8^+^ T cells; A5 mostly at 48 h and DI primarily at 72 h (Fig. S1m,n). Finally, none of the compounds affected the frequency of live CD4^+^ or CD8^+^ T cells (Fig. S1a–h, respectively).

### SDH activity is essential for T cell cytokine production

**Figure 4:**
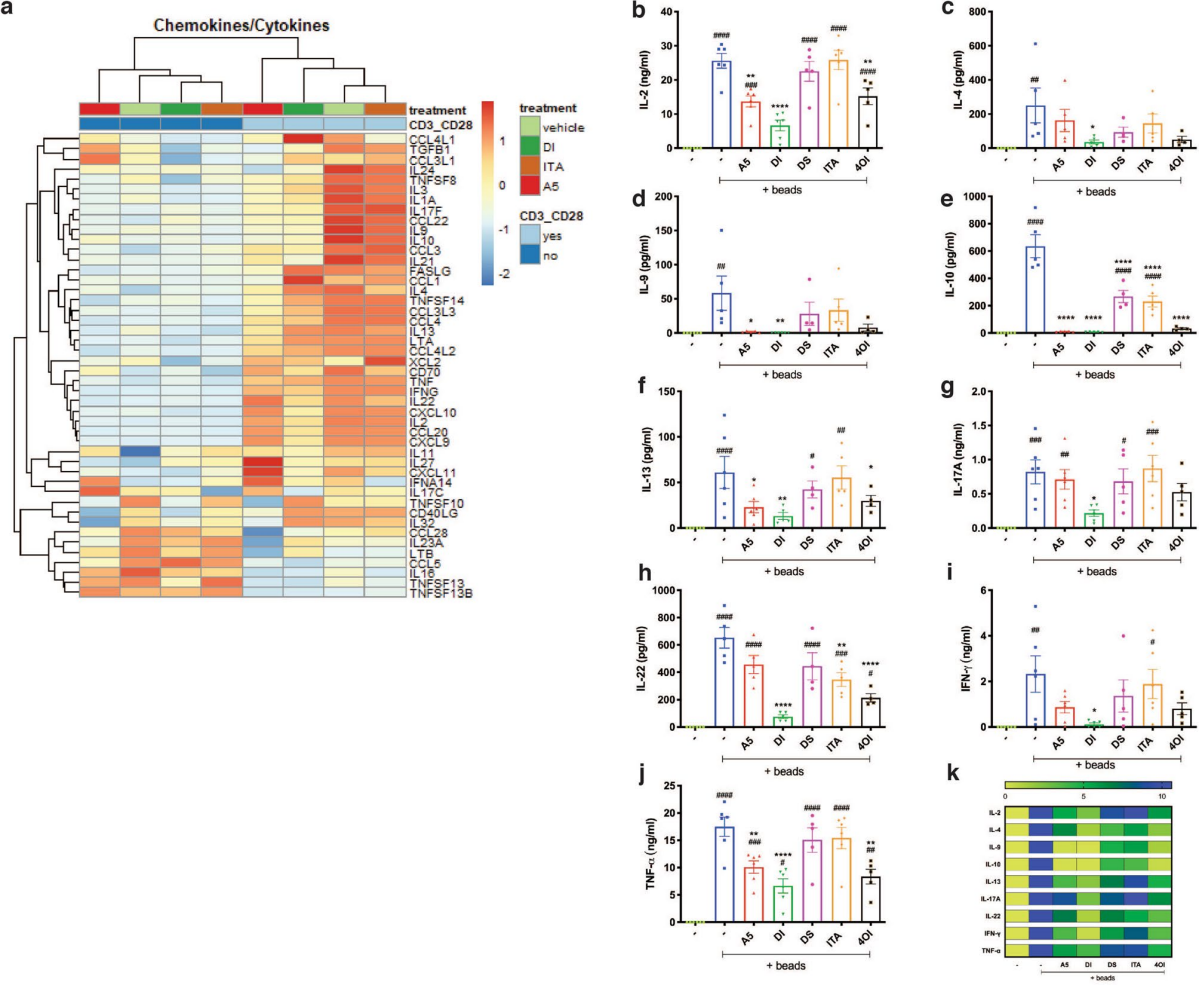
SDH activity is essential for T cells cytokine production. (**a**) Heat map with Z-score of cytokines- and chemokines-related genes from Affimetrix gene expression array performed on (pooled RNA from n = 3 independent experiments) resting and beads-activated CD4^+^ T cells exposed to A5 (20 μM), DI (250 μM), or ITA (1 mM). (**b**–**j**) Secreted cytokines by CD4^+^ T cells after 24 h activation and drug-treatment (n = 4–6 independent experiments, with n = 2 technical replicates) measured by MSD and ELISA. All charts show mean and ± s.e.m. (**k**) Concentration of cytokines were normalized to each activated untreated control and are displayed in a color-scaled heat-map. P values are calculated using one-way ANOVA test followed by Dunnet’s test to different control groups: (#) to the unstimulated and untreated control, and (*) to the activated untreated control. # or *P < 0.05, ## or **P < 0.01, ### or ***P < 0.001, #### or ****P < 0.0001.

In order to investigate whether SDH inhibition would compromise cytokine and chemokine expression, we exposed purified CD4^+^ T cells to A5, ITA or DI, for 24 h during the early activation. As expected, activation of CD4^+^ T cells induced expression of a wide range of cytokines and chemokines (Fig. 4a). The analysis revealed that none of the compounds altered the expression of cytokine and chemokine genes in unstimulated cells. In contrast, expression of many of the induced cytokines and chemokines in activated cells was modulated (Fig. 4a). In details, the data showed a strong immunomodulatory capacity mediated by A5, and DI, compared to vehicle and ITA-treated samples (Fig. 4a). The effect on expression of the important inflammation mediators IFNG and TNFA were additionally assayed by qPCR, and the analysis confirmed that A5 and DI strongly inhibit the expression of both, while 4OI only affected expression of IFNG, ITA had no effect on expression of either gene (Fig. S4a,b).

To evaluate the importance of SDH fitness on CD4^+^ T cell functionality, we next measured production of selected cytokines by ELISA and MSD. This time we also used ITA and its derivatives to explore their effect upon cytokine production by T cells. The analysis revealed that DI potently inhibited secretion of all targeted cytokines (Fig. 4b–j). In contrast, A5 only significantly inhibited IL-2, IL-9, IL-10, IL-13, TNF-α (Fig. 4b,d–f,j). In addition, although not significant, we also observed a tendency of A5-mediated reduction of IL-22 and IFN-γ secretion (Fig. 4h,i), whereas no effect was observed on IL-4 and IL-17A (Fig. 4c,g). Whether this effect was exclusively mediated by the increase of the intracellular succinic acid was unclear, as exogenous supplementation of succinate in the form of dimethyl-succinate, did not elicit the same result. 4OI partly overlapped with the effect of A5, and significantly inhibited secretion of IL-2, IL-10, IL-13, IL-22, and TNF-α (Fig. 4b,e,f,h,j). Surprisingly, ITA impaired the secretion of IL-10 family members such as IL-10, and IL-22 (Fig. 4e,h). We additionally plotted the cytokine concentration data in a colour-scaled heat-map where values were normalized to the activated untreated control, for a better visualisation of the differences induced by the activation stimulus and the reduction due to each individual compound (Fig. 4k). Taken together, these data show that A5-mediated SDH inhibition impairs T cell cytokine production, indicating that TCA and the electron transport chain (ETC) are important for proper T cell activation and functionality. Furthermore, ITA and its derivatives individually modulate T cell cytokine production differently, likely due to differences in activity towards different molecular targets or mechanism (see discussion).

### SDH inhibition alters activated T cells cytokine production

**Figure 5:**
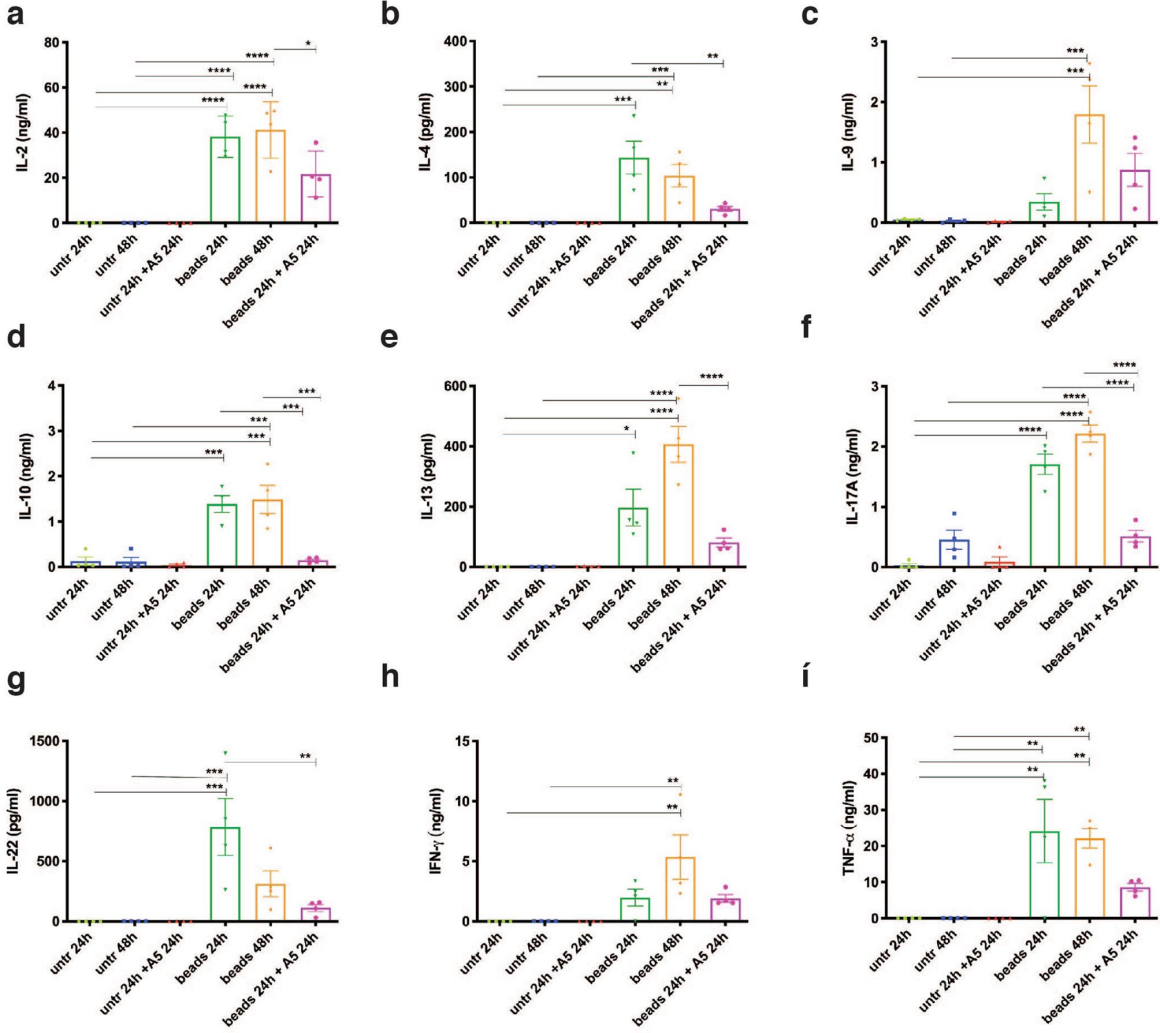
SDH inhibition affect cytokine production after T cell activation. (**a**–**i**) Secreted cytokines by CD4^+^ T cells after activation and compound-treatment (A5, 20 μM) measured by MSD and ELISA (n = 4 independent experiments, with n = 2 technical replicates). All charts show mean and ± s.e.m. P values are calculated using one-way ANOVA with Tukey multiple comparisons. *P < 0.05, **P < 0.01, ***P < 0.001, ****P < 0.0001.

To further investigate the extent of SDH inhibition in influencing T cells cytokine release, we pre-activated CD4^+^ T cells for 48 h and treated them with A5 for the last 24 h, after the initial T cell activation and metabolic switch have already occurred. As expected, resting CD4^+^ T cells were not perturbed by A5, and their cytokine profile was dormant (Fig. 5a–i). In contrast, activated T cells showed an increased release of all the assayed cytokines over time and especially for the IL-9, IL-13, IFN-γ the fold change was higher after 48 h activation, compared to 24 h. Furthermore, the cells exposed to A5 homogenously and, in most cases, significantly reduced the release of all tested cytokines (Fig. 5a–i). This highlights the importance of SDH fitness, not only during the early steps of T cell activation but also at later stages, and for all T cell subsets.

### Mitochondrial respiration is essential for proper T cell activation

**Figure 6:**
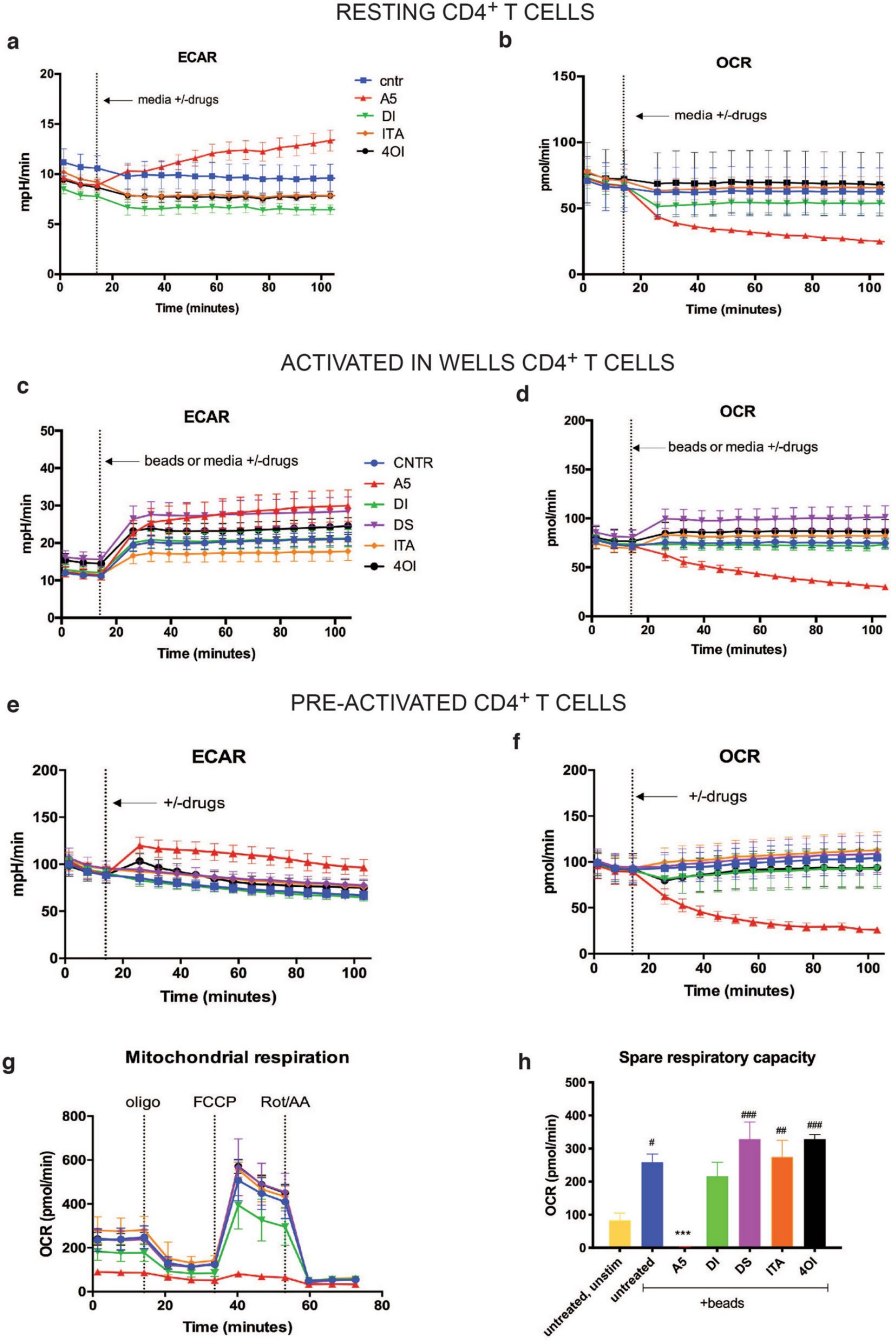
Mitochondrial respiration is pivotal for a proper T cell activation. Seahorse analysis of ECAR and OCR in (**a**, **b**) resting, (**c**, **d**) activated in wells, (**e**–**h**) and pre-activated CD4 + T cells. (**g**, **h**) Mitochondrial stress assay performed with oligomycin, FCCP, and rotenone/antimycin A on pre-activated and pre-treated CD4^+^ T cells. (**a**–**f**) n = 9 independent experiments, (**g**, **h**) n = 3 independent experiments, all with n = 3 technical replicates. Charts show mean and ± s.d, (**a**–**g**) or s.e.m. (**h**). As previously, we treated the cells with: A5 (20 μM), DI (250 μM), DS (1 mM), ITA (1 mM), 4OI (125 μM). P values are calculated using one-way ANOVA test followed by Dunnet’s test to different control groups: (#) to the unstimulated and untreated control, and additionally (*) to the activated untreated control. # or *P < 0.05, ## or **P < 0.01, ### or ***P < 0.001, #### or ****P < 0.0001.

To achieve a deeper understanding of the metabolic impact of SDH inhibition in real-time, we performed seahorse analysis on either in-well resting (Fig. 6a,b), in-well activated and compound treated (Fig. 6c,d), or 24 h-preactivated in vitro and in-well compound treated (Fig. 6e,f) purified CD4^+^ T cells. Overall, the data indicated that only A5 affected mitochondrial oxygen consumption, reducing the OCR values in all three analysed CD4^+^ T cell conditions (Fig. 6b,d,f). As a compensatory effect, the ECAR values (corresponding to extracellular acidification) increased as glycolysis was employed to provide energy for CD4^+^ T cells treated with A5 (Fig. 6a,c,e). This latter aspect was more prominent in resting and pre-activated CD4^+^ T cells (Fig. 6a,e). Furthermore, a mitochondrial stress assay (Fig. 6g) on 24 h-preactivated and compound-treated cells showed that A5-mediated SDH inhibition disrupted oxygen consumption and significantly reduced T cell spare respiratory capacity (SRC) compared to the activated untreated control (Fig. 6h). Similar effects of A5 on OCR and ECAR were observed in purified human CD8^+^ T cells (Fig. S4). Also, the SRC was significantly reduced by A5 in CD8^+^ T cells compared to the activated untreated control (Fig. S4d). Furthermore, neither ITA nor its derivatives changed ECAR or OCR values in both CD4^+^ (Fig. 6) and CD8^+^ T cells (Fig. S4) suggesting that T cell metabolism is inert towards these compounds in the evaluated concentration.

## Discussion

Activation of T cells changes their metabolic requirements. Thus, non-activated T cells are quiescent and requires only low-level metabolic activity to fuel migration and homeostatic functions. In contrast, activation, and the transition from naive to effector lymphocyte, greatly alters cellular metabolic demands, as these cells require both ATP and biosynthetic components to fuel proliferation, migration, and subset differentiation^1,2,33^.

Activation-induced metabolic reprogramming is a switch towards highly glycolytic metabolism, and comparatively low rates of OXPHOS, preferentially secreting glucose-liberated carbon as lactate^30,33^. In the mitochondria SDH couples two major pathways essential for OXPHOS, namely TCA and ETC. Indeed, SDH is responsible for conversion of succinate into fumarate in the TCA, as electrons acceptor complex II in OXPHOS, donating two hydrogens as part of the ETC. Succinate fuels isolated mitochondria but have recently also been implicated in many other important cellular processes, including epigenetic modulation, cancer cell metabolism, mediation of hypoxic responses, ROS metabolism, endo- and paracrine modulation, posttranslational modifications, and signalling during inflammation^17,19,20^.

The contribution of the TCA in T cell function and differentiation has been lately revaluated but is still not fully understood^34–36^. It has previously been demonstrated that the proliferative capacity of both CD4^+^ and CD8^+^ T cells is more sensitive towards inhibition of OXPHOS (complex I) rather than glycolysis. In addition, impairment of glycolysis has a stronger effect on biomass increase in CD4^+^ T cells, than in CD8^+^ T cells^30^.

Here we present new data showing that proliferation, apoptosis and mitochondrial potential are strongly influenced by SDH/complex II fitness in activated human T cells. Indeed, SDH inhibition led to a strong impairment of proliferation, neutralization of mitochondrial membrane potential, and a decrease in cytokine secretion, which are all integral parts of T cell functionality.

In support, it was recently shown that the complex II-inhibitor A5 could reduce IFN-γ production in mouse Th1 cells, and that inhibition of complex I and III, but not II, impaired Th1, Th2, and Th17 proliferation^15^. Here we have demonstrated that SDH/complex II inhibition in human T cells during early activation, led to a strong reduction of cytokine and chemokine expression (as showed in Fig. 4a), and lower secreted protein levels of IL-2, IL-4, IL-9, IL-10, IL-13, IL-17A, IL-22, TNF-α, and IFN-γ (as showed in Fig. 4b–i). Furthermore, SDH inhibition at later stages of CD4^+^ T cell activation also led to a strong reduction of all tested cytokines, highlighting the importance of SDH in T cell functionality. As expression of most of the Th1, Th2, Th17, Th22, Treg cytokines analysed were impaired, our data indicated that all CD4^+^ T cell effector subsets are affected by SDH inhibition (Fig. 5).

In addition, we demonstrated that blocking complex II blunted the spare respiratory capacity of CD4^+^ and CD8^+^ T cells (Fig. 6g,h and Fig. S4c,d) indicating that, under increased work or stress conditions, T cells could not generate energy for long-term survival and functionality. This further supports the importance of TCA both in generating NADH and succinate to fuel complex I and complex II, respectively, and the crucial role of ETC in sustaining the metabolic demands that occur in activated human CD4^+^ and CD8^+^ T cells.

Moreover, we have observed that the amino acid metabolism is not perturbed in SDH-inhibited CD4^+^ T cells. This is in contrast to what was shown in paraganglioma tumour cells^37^ indicating that the underlying effects mediated by SHD inhibition may be cell- and/or metabolism-dependent.

Our data additionally shed light on the effect of itaconic acid on human T cells. Briefly, TCA-derived intermediate itaconic acid, which is produced via citrate accumulation in activated dendritic cells and M1 macrophages, has been considered as an immune-regulatory molecule in macrophages via inhibition of SDH^24–26^. Because itaconic acid is a α,β-unsaturated carboxylic acid, it was initially thought to be cell-impermeable, and cell-permeable analogues were synthesised to satisfy the burgeoning request by the itaconate biology research field. The synthesis included esterification of the carboxylic groups and the production of some cell-permeable derivatives, such as DI and 4OI^38^. Those two derivatives have already been used for immunological studies and have opened new perspectives as potential therapeutics in inflammatory diseases, especially DI as treatment for psoriasis^37^ and 4OI for systemic lupus erythematosus^39^. These promising results led us to wonder about their potential upon human T cells and to explore their effects since the state-of-the-art currently lacks knowledge.

Originally, some studies showed that DI was not metabolized into itaconate intracellularly, but did induce an increased itaconate biosynthesis, possibly due to the electrophilic effect or, alternatively, through a receptor-mediated pathway^22,32^. Very recent findings have revealed that exposing both resting and activated murine bone-marrow derived macrophages (BMDMs) to physiologic, non-esterified itaconate, leads to an increase of its intracellular level after 3 h, while neither DI nor 4OI induce the same, although being absorbed by the cells^27^.

Here, we present for the first time the endo-metabolome analysis of human CD4^+^ T cells after activation and exogenous exposure to ITA, DI and 4OI. Our results revealed that ITA was internalized (and readily isomerized into citraconic acid) (Fig. S2e) but surprisingly this did not induce succinate accumulation, indicating that SDH enzymatic activity was not inhibited. On the other hand, DI and 4OI, which have been previously studied in macrophages^27,40,41^, were not found intracellularly. Furthermore, neither of these compounds led to SDH inhibition, and subsequent increase in intracellular succinate concentration (Fig. 2a–c, Fig S2c,f), although 4OI did increase itaconic and crotonic acid levels. Our data are supported by the latest findings where the ester-derivatives of itaconate did not inhibit SDH enzymes in vitro and only itaconate (and marginally 4OI) induced the intracellular accumulation of succinate^27^. Interestingly, we showed that the addition of exogenous succinate to the culture media, intended to mimic a massive increase of succinate, did not raise intracellular succinate levels, as shown by the endo-metabolome analysis and succinic acid quantification (Fig. 2a–c, Fig. S2d); instead succinate was likely absorbed, modified by cellular esterases and partially entered the TCA (Fig. 2a–c, and Fig. S2d).

Here we also show that both DI and 4OI, affected surface expression of the CD4^+^ and CD8^+^ T cell activation markers CD69 and CD25 (Fig. 3 and Fig. S1), and modified CD4^+^ T cell cytokine production (Fig. 4) with differing degrees of potency. We found that their effect was presumably due to activation of nuclear factor erythroid 2-related factor 2 (NRF2), involved in oxidative stress responses, as we observed DI and 4OI inducing an increased expression of classical NRF2-target genes HMOX1, GCLM, NOQ1 in human CD4^+^ T cells (Fig. S3c–e), as also shown in resting and activated macrophages^27^. As previously shown^26,41^, DI directly activates NRF2 and thereby limits inflammation in mouse and human macrophages, while 4OI activates NRF2 indirectly via alkylation and disassociation of its inhibitor Kelch Like Associated Protein 1 (KEAP1) resulting in promoted NRF2-mediated transcriptional activity and downstream anti-oxidant and anti-inflammatory properties by reducing expression of TNF-α, IL-1β, and IL-6^39,41^.

In human CD4^+^ T cells, KEAP1 gene expression levels were significantly induced by the anti-CD3/CD28 stimulation*.* Treatment of CD4^+^ T cells with A5 did not affect expression of any of the classic NRF2-target genes (Fig. S3c–e) but instead A5 significantly reduced expression of both NFE2L1 (gene encoding for NRF1 protein), NFE2L2 (NRF2 protein), and KEAP1 (Fig. S3f–h) suggesting that mitochondrial activity, through TCA and/or ETC, regulates expression of these genes. In addition, we found that neither DI nor 4OI altered the expression of NFE2L1, NFE2L2 and KEAP1 compared to the stimulated control (Fig. S3f–h).

Interestingly, treatments with itaconate-derivatives DI and 4OI were lately explained to limit IFN-β release after lipopolysaccharide (LPS) stimulation in murine macrophages^40,41^. While itaconate inhibited IL-1β (but not TNF-α and IL-6) and surprisingly induced IFN-β release, both DI and 4OI did not alter TNF-α but inhibited IL-6, IL-1β, and IFN-β^27,41^. These effects were also demonstrated as SDH-independent, underscoring that itaconate and its derivatives exhibit a mechanistically distinct action on murine BMDMs^27^, and now also confirmed on human T cells.

In conclusion, we have shown that T cells despite switching to aerobic glycolysis when activated, still rely on mitochondrial metabolism via TCA and ETC for proliferation and functional responses. Furthermore, our data have demonstrated the inefficacy of itaconic acid to influence T cell biology, revealing a selective activity towards DCs and macrophages. Finally, our data indicated that the different effects induced by the ester-derivative of itaconate, such as DI and 4OI, are not mediated via inhibition of SDH, but likely via effects on the NRF2/KEAP1 pathway.

## Materials and methods

### Chemicals

Atpenin A5 (Caymann Chemical, CAS 119509-24-9), dimethyl-succinate (Sigma-Aldrich, CAS 106-65-0), itaconic acid (Sigma-Aldrich, CAS 97-65-4), dimethyl-itaconate (ChemCruz, CAS 617-52-7), 4-octy-itaconate (Sigma-Aldrich, CAS 3133-16-2), dimethyl-succinate (Sigma-Aldrich, 100-65-0).

### PBMC isolation and CD4^+^/CD8^+^ T cells enrichment

Peripheral blood mononuclear cells (PBMCs) were isolated from healthy donor’s buffy coats obtained from Rigshospitalet, Copenhagen, Denmark in accordance with the Declaration of Helsinki. Written informed consent was obtained from blood donors at the Department of Clinical Immunology, University Hospital Rigshospitalet, Copenhagen and used without the possibility to identify case specific information. The ethical committee, Region H, Capital Region of Denmark, approved the use of these buffy coats for research that was carried out in accordance with the approved guidelines. Lymphoprep density-gradient centrifugation (Stemcell technologies) was used and the isolated cells taken for the following steps, as needed. CD4^+^ T cells and CD8^+^ T cells were separately enriched using Dynabeads Untouched Human CD4^+^ or CD8^+^ T cells kit (Invitrogen, cat. 11346D or 11348D, respectively) following the manufacturer’s instructions. When specified, T cells were stimulated in vitro with Dynabeads Human T activator CD3/CD28 for T cells expansion and activation (Thermo Fisher, cat # 11131D), (5:2 cell:beads ratio).

### Flow cytometry

Cell surface staining was performed in FACS-PBS (PBS + 1% FBS + 0.02% NaN_3_) or Brilliant Stain Buffer (BD Bioscience, #563794) when required, using primary anti-human conjugated antibodies. Dead cells were excluded using propidium iodide (eBioscience, #MBS500PI) and annexin V (FITC, BioLegend, #640945). Annexin V staining was conducted in Annexin V Binding Buffer (BD Bioscience, #51-66121E). All antibodies were purchased at BD Biosciences: CD4 BV711 (SK3, 563028), CD8 BV421 (RPA-T8, 562428), CD25 BUV395 (M-A251, 740290), CD69 PE-Cy7 (FN50, 561928). MitoTracker Red CMXRos (ThermoFisher, M7512) was used for detecting mitochondrial membrane potential, following the manufacturer’s instruction. All flow cytometric analyses were performed using a 5 laser BD Fortessa (Becton Dickinson) at the Core Facility for Flow Cytometry (CFFC), University of Copenhagen. Flow cytometry data were visualized and analysed using FlowJo 8 or 10 (TreeStar) software.

### RNA purification, cDNA synthesis, and qPCR

Total RNA was isolated using RNeasy Mini Kit (Qiagen) and cDNA was transcribed using the High Capacity cDNA Reverse Transcription Kit followed by PCR analysis using TaqMan Gene Expression Assay method. All TaqMan probes were purchased from LifeTechnologies (POLR2A Hs00172187_m1, HMOX1 Hs01110250_m1, GCLM Hs00978072_m1, NQO1 Hs01045993_g1, S100A7A Hs00752780_s1, LCN1 Hs06650119_g1, ATF3 Hs00231069_m1, KEAP1 Hs00202227_m1, NFKBIZ Hs00230071_m1, NRF1 Hs00602161_m1, NFE2L2 Hs00975961_g1, TNFSF2 Hs00174128_m1, IFNg Hs00989291_m1). Quantitative polymerase chain reaction (qPCR) was performed using the TaqMan assay from ThermoFisher Scientific in accordance with the manufacturer’s instructions, and the samples were analyzed on a LightCycler480 II instrument (Roche). Results are presented as relative quantity to the control sample determined by the ddCt method, using POLR2A as reference gene.

### RNA isolation for Affymetrix microarray analysis

RNA was isolated and purified using the RNeasy kit (Qiagen) according to the manufacturer’s instructions, then quantified using a spectrophotometer (NanoDrop, Wilmington, DE). Equal concentrations of total RNAs derived from the three experiments were pooled, each treatment with its own corresponding control. Sample labeling and microarray hybridization were performed according to the manufacturer’s instructions (Affymetrix). Global gene expression analysis was conducted using Affymetrix GeneChip Human Transcriptome Array HTA 2.0 covering > 285,000 full-length transcripts (performed by Rigshospital, Copenhagen). Array data were summarized with the RMA (Robust Multichip Average algorithm) and genes with a signal intensity less than four in all samples were filtered out (cut-off: 1.5-fold change). Heatmaps, unsupervised hierarchical clustering and principal component analysis were performed in R Bioconductor.

### ELISA and MSD analysis

T cells supernatants were previously stored at − 80 °C immediately after cells harvesting. IL-2, IFN-γ, TNF-α were measured by DuoSet ELISA (R&D kits), following the manufacturer’s instructions. For all the other cytokines simultaneous quantification was carried out on a customized U-PLEX assay platform with the electrochemiluminescence immunoassays technology (MSD), and data was acquired via Meso QuickPlex SQ120 at the CFFC.

### Seahorse analysis

Oxygen consumption rate (OCR, pmoles/min) and extracellular media acidification rate (ECAR, mpH/min) were measured using a XF96 extracellular flux analyzer (Agilent, Seahorse Bioscience) at the CFFC. Cells were washed three times, plated at 0.7 or 1 × 10^6^ cells (CD8^+^ and CD4^+^ T cells, respectively) per well in a 96-well Seahorse assay plate, precoated with Cell-Tack Cell and tissue adhesive (Corning). OCR and ECAR were measured after equilibration at 37 °C for 30 min before assay. The Mitostress tests were performed on T cells after 24 h beads-induced activation and treatments in vitro. Cells were plated in Agilent Seahorse XF RPMI medium with 10 mM glucose, 1 mM sodium pyruvate and 2 mM glutamine. Mitochondrial stress assays were performed upon cell treatment with oligomycin (1 mM), FCCP (0.5 mM), rotenone/antimycin A (1 μM), according to the manufacturer’s instructions. All the experiments were performed in order to collect from 3 to 9 biological replicates, as independent experiments, and each time three technical replicates were analyzed.

### Metabolome extraction and LC–MS analysis

Initially, 3 × 10^6^ cells were pelleted (300*g* for 2 min at room temperature) and the medium aspired followed by immediate addition of 1.5 mL ice-cold 80% MeOH and snap-frozen in liquid nitrogen. The samples were subsequently thawed on ice, vortexed for 30 s before being snap-frozen again and the procedure repeated for a total of three cycles. Finally, any undissolved fractions were pelleted (12,000*g* for 10 min at 0 °C) and the supernatant dried under a light flow of N_2_ before the samples were resuspended in 30 μL 50% acetonitrile (pH 9) and diluted two times in 10 mM ammonium acetate in 90% acetonitrile (pH 9). Analysis of the intracellular metabolites was performed by MS-Omics. Overall, the LC–MS method was modified from Hsiao et al*.*^42^ using a Thermo Scientific Vanquish LC coupled to a Thermo Q Exactive HF MS with a heated electrospray ionization interface operated in negative and positive ionization mode. For the untargeted analysis peak areas were extracted using Compound Discoverer (vers: 3.0.0.294, Thermo Fisher Scientific Inc.), while the targeted analysis was conducted using TraceFinder (vers 4.1, Thermo Fisher Scientific Inc.).

### Succinate quantification

CD4^+^ T cells were seeded at 2 × 10^6^/well, treated and activated for 24 h with beads (5:2 cell:beads ratio), then harvested and used for succinate quantification using the succinate colorimetric assay (Abcam, ab204718) following the manufacturer’s instructions.

### Statistical analysis

Statistical analysis was performed with Prism 8 software (Prism 8.4.2 (464) for OS X) (GraphPad Software, San Diego, California USA, www.graphpad.com). Data are expressed as mean ± s.e.m. or s.d, as specified in each figure legend. *P* values were calculated using one-way or two-way analysis of variance (ANOVA) for multiple comparison of variables. Dunnett’s multiple comparisons test was used to compare the means to a control mean, unless differently specified in the figure legend. A confidence interval of 95% was used for all statistical tests. Sample sizes were determined based on the experiment type and standard practice in the field.

## Supplementary Information

Supplementary Figures.
