# Publisher Correction: Inhibition of succinate dehydrogenase activity impairs human T cell activation and function

**DOI:** 10.1038/s41598-021-88184-w

**Published:** 2021-04-19

**Authors:** Claudia Nastasi, Andreas Willerlev-Olsen, Kristoffer Dalhoff, Shayne L. Ford, Anne-Sofie Østergaard Gadsbøll, Terkild Brink Buus, Maria Gluud, Morten Danielsen, Thomas Litman, Charlotte Mennè Bonefeld, Carsten Geisler, Niels Ødum, Anders Woetmann

**Affiliations:** 1 LEO Foundation Skin Immunology Research Center & Department of Immunology and Microbiology, University of Copenhagen, Panum Institute, The Maersk tower, 07.12.76, Blegdamsvej 3C, 2200 Copenhagen, Denmark; 2 MS-Omics, Vedbæk, Denmark; 3 Department of Immunology and Microbiology, University of Copenhagen, Copenhagen, Denmark; 4 LEO Pharma A/S, Ballerup, Denmark

Correction to: *Scientific Reports* 10.1038/s41598-020-80933-7, published online 14 January 2021

The Author Contributions section in this Article is incorrect.

“A.W.-O. and C.N. designed the research. C.N. performed experiments, analysed and interpreted the data, made the figures, and wrote the original draft of the paper. A.W.-O. performed microarray analysis. K.D. and M.D. performed metabolome analysis. C.N., A.W.-O., K.D., S.L.F, A.S.Ø.G., T.B.B., M.G., M.D., C.M.B., C.G., T.L., N.Ø. and A.W. reviewed and edited the manuscript. A.W.-O. acquired the funding.”

should read:

“A.W. and C.N. designed the research. C.N. performed experiments, analysed and interpreted the data, made the figures, and wrote the original draft of the paper. A.W.-O. performed microarray analysis. K.D. and M.D. performed metabolome analysis. C.N., A.W–O., K.D., S.L.F, A.S.Ø.G., T.B.B., M.D., C.M.B., C.G., T.L., N.Ø. and A.W. reviewed and edited the manuscript. A.W. acquired the funding.”

