# Supplementary material for: Inhibition of succinate dehydrogenase activity impairs human T cell activation and function
Source: Sci Rep. 2021 Jan 14;11:1458. doi: 10.1038/s41598-020-80933-7 (PMC7809054; doi:10.1038/s41598-020-80933-7)
Supplement: Supplementary file 1 — Supplementary Figures. [file 41598_2020_80933_MOESM1_ESM.docx]

**Inhibition of succinate dehydrogenase activity impairs human T cell activation and function**

Claudia Nastasi^1^, Andreas Willerlev-Olsen^1^, Kristoffer Dalhoff^2^, Shayne L. Ford^1^, Anne-Sofie Østergaard Gadsbøll^1^, Terkild Brink Buus^1^, Maria Gluud^1^, Morten Danielsen^2^, Thomas Litman^3,4^, Charlotte Mennè Bonefeld^1^, Carsten Geisler^1^, Niels Ødum^1^, and Anders Woetmann^1*^.

^1^LEO Foundation Skin Immunology Research Center, Department of Immunology and Microbiology, University of Copenhagen, Copenhagen, Denmark. ^2^MS-Omics, Vedbæk, Denmark. ^3^Department of Immunology and Microbiology, University of Copenhagen, ^4^LEO Pharma A/S, Ballerup, Denmark.

*****Corresponding author: Anders Woetmann, LEO Foundation Skin Immunology Research Center, Department of Immunology and Microbiology, University of Copenhagen, Panum Institute, The Maersk tower, 07.12.76, Blegdamsvej 3C, DK-2200, Copenhagen, Denmark. E-mail: awoetmann@sund.ku.dk.

**Supplemental material**

**
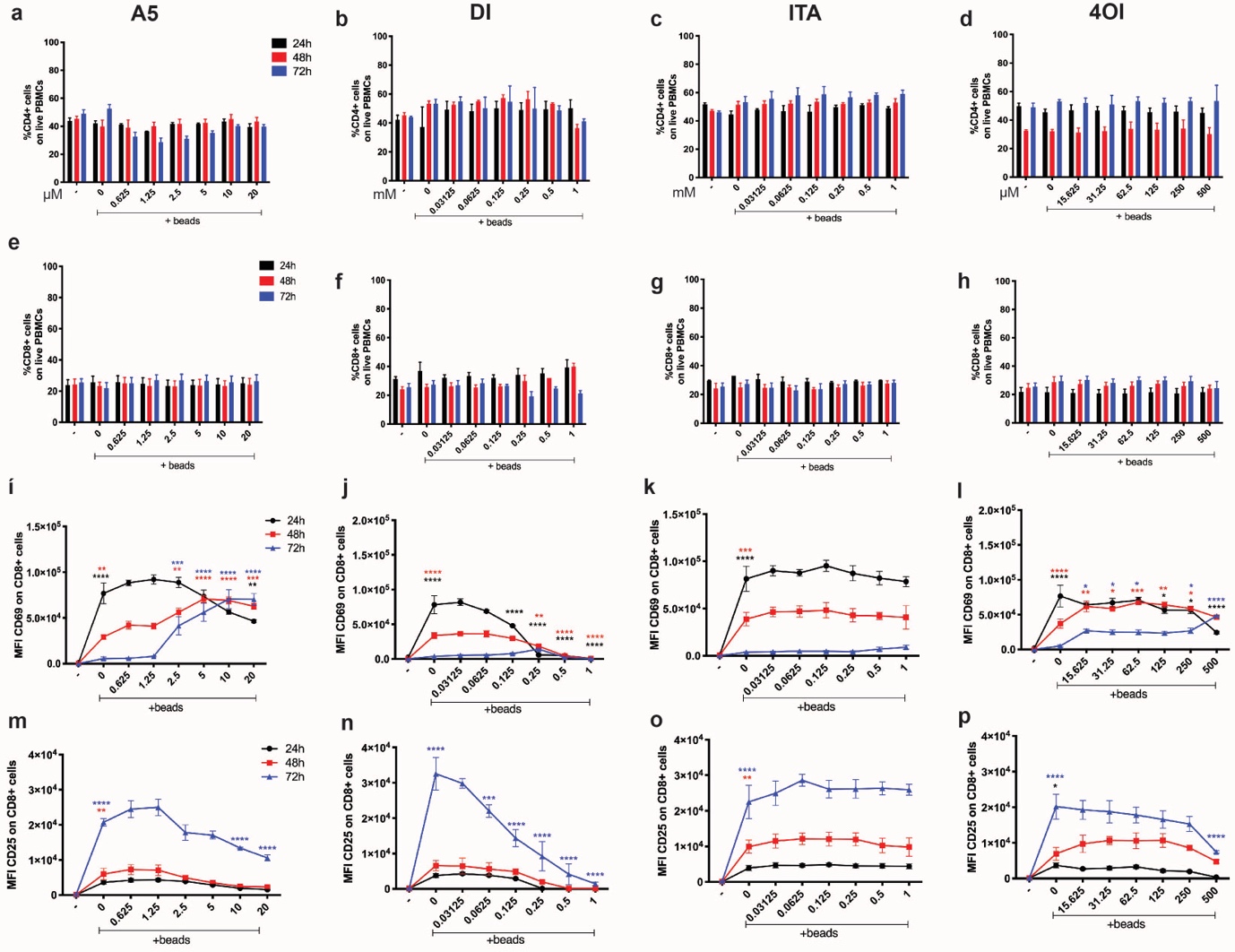
**

**Supplemental Figure S1. Flow cytometry analysis of CD4^+^ and CD8^+^ T cells among live PBMCs and analysis of the surface activation markers CD25 and CD69 upon CD8^+^ T cells.** **(a-d)** Frequencies of human CD4^+^ T cells and **(e-h)** CD8^+^ T cells among live PBMCs. Cells exposed to **(a,e,I,m)** A5, **(b,f,j,n)** DI, **(c,g,k,o)** ITA, and **(d,h,l,p)** 4OI in a concentration gradient and analysed after 24h (black), 48h (red), and 72h (blue) by flow cytometry. All charts show mean and ± s.e.m. Mean Fluorescence Intensity (MFI) ± s.e.m. are shown for CD25 and CD69 expression levels (n=3 independent experiments). P values are calculated using two-way ANOVA. *P < 0.05, **P < 0.01, ***P < 0.001, ****P < 0.0001.

**
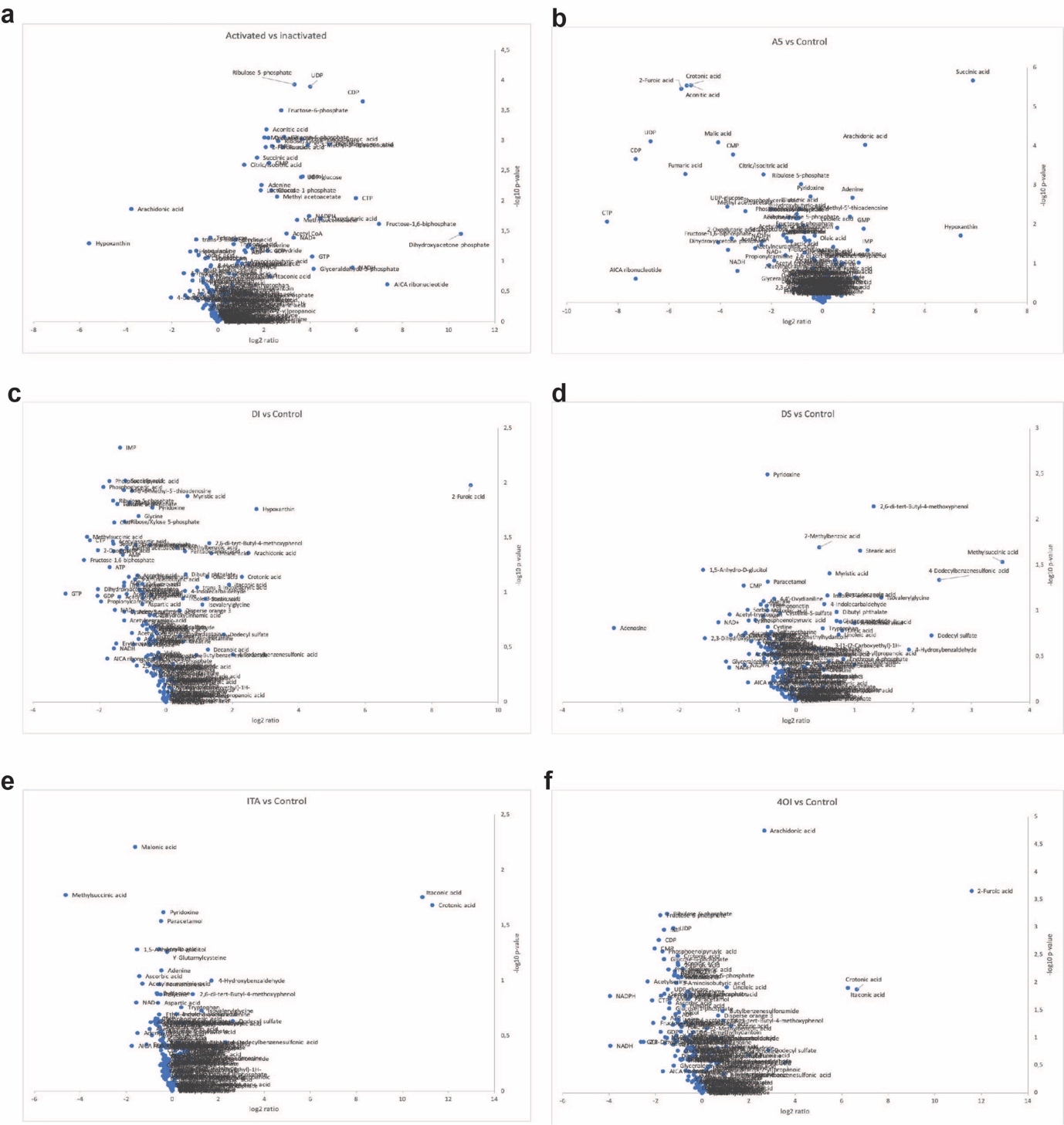
**

**Supplemental Figure S2. Volcano plots of CD4^+^ T cell endo-metabolites.** Cellular metabolites measured by liquid-chromatography-mass spectrometry. Comparisons between CD4^+^ T cells **(a)** activated versus inactivated, **(b)** activated vs A5-treated (20uM), **(c)** activated vs DI-treated (250uM), **(d)** activated vs DS-treated (1mM), **(e)** activated vs ITA-treated (1mM), and **(f)** activated vs 4OI-treated (125uM), (n=3 independent experiments).

**
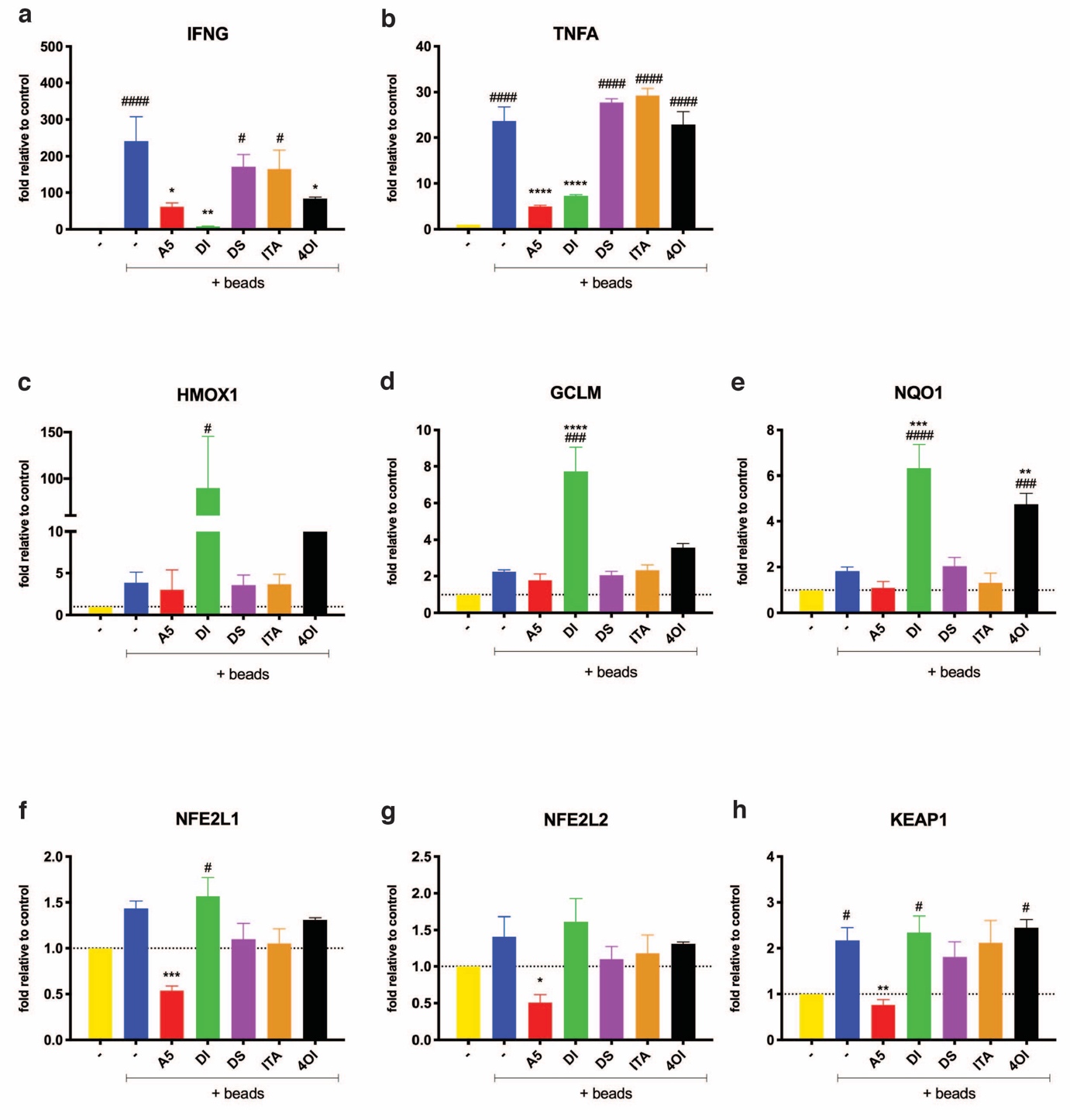
**

**Supplemental Figure S3. Real-time quantitative PCRs (RT-qPCRs).** Gene expression analysis on human CD4^+^ T cells was assessed for (**a,b)** cytokines IFNG and TNFA, **(c-e)** Nrf2 target genes, **(f-h)** genes encoding for transcription factors such as NFE2L1, NFE2L2 and KEAP1 (n=3-6 independent experiments, with n=3 technical replicates). As previously, we used the following concentrations: A5 (20uM), DI (250uM), DS (1mM), ITA (1mM), 4OI (125uM). Results are presented as relative quantity to the control sample determined by the ddCt method, using POLR2A as reference gene and unstimulated untreated sample as calibrator. P values are calculated using one-way ANOVA test followed by Dunnet’s test to different control groups: (#) to the calibrator (unstimulated and untreated control), and additionally (*) to the activated untreated control. # or *P < 0.05, ## or **P < 0.01, ### or ***P < 0.001, #### or ****P < 0.0001.

**
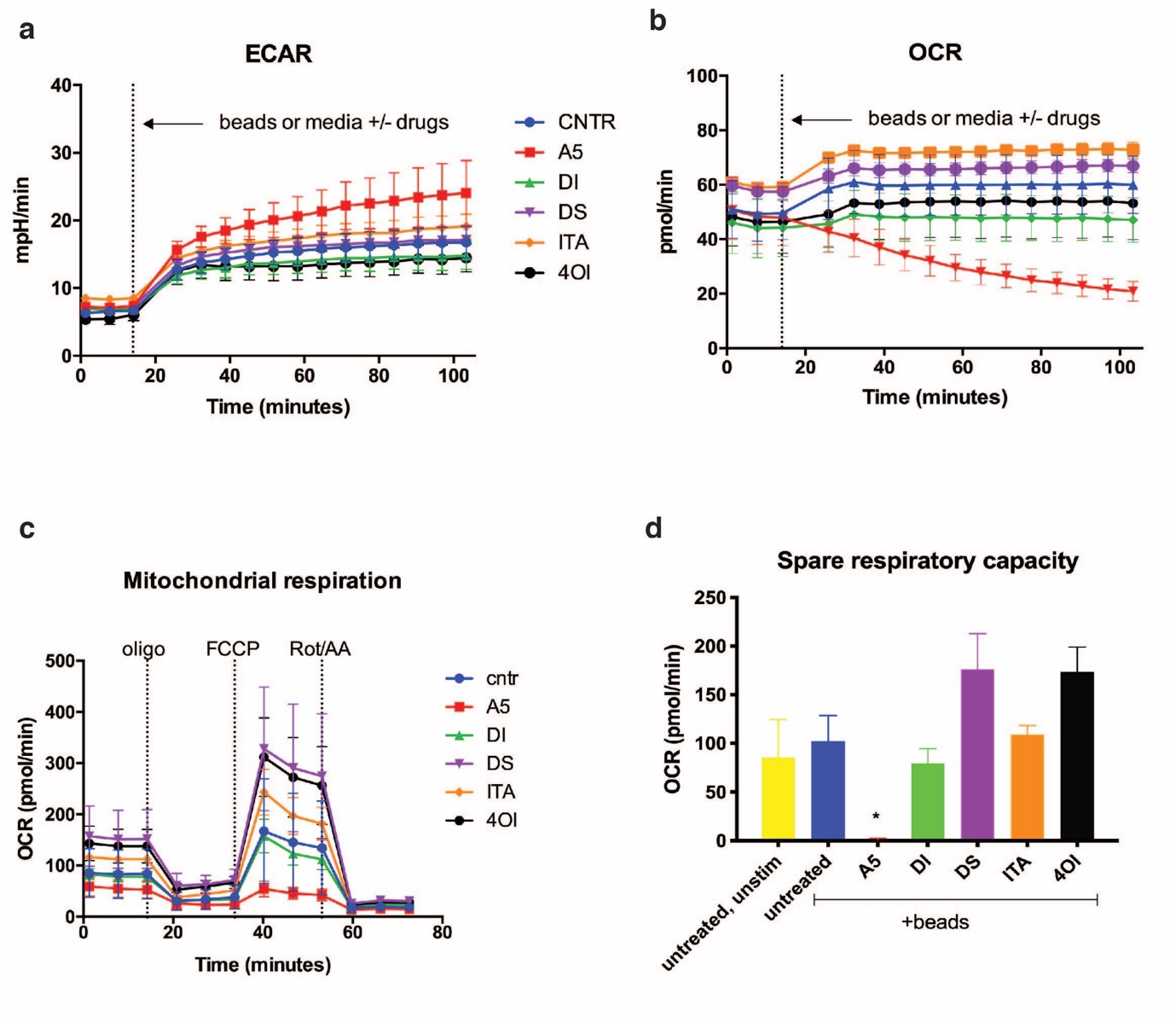
**

**Supplemental Figure S4. Mitochondrial respiration is essential for a proper CD8^+^ T cell activation.**

Seahorse analysis of OCR and ECAR in activated in wells **(a-b)** (n=3 independent experiments, and n=3 technical replicates). **(c)** Mitochondrial stress assay performed with oligomycin, FCCP, and rotenone/antimycin A on pre-activated and pre-treated CD4^+^ T cells (n=3 independent experiments, with n=3 technical replicates). Charts are representative of mean and ± s.d, **(a-b)** or s.e.m. **(c-d)**. As previously, the following concentrations have been used: A5 (20uM), DI (250uM), DS (1mM), ITA (1mM), 4OI (125uM). P values are calculated using one-way ANOVA test followed by Dunnet’s test to the activated untreated control. *P < 0.05, **P < 0.01, ***P < 0.001, ****P < 0.0001.
